# Radiotherapy in the management of gist: state of the art and new potential scenarios

**DOI:** 10.1186/s13569-016-0065-z

**Published:** 2017-01-10

**Authors:** L. Gatto, M. Nannini, M. Saponara, V. Di Scioscio, G. Beltramo, G. P. Frezza, G. Ercolani, A. D. Pinna, A. Astolfi, M. Urbini, G. Brandi, G. Biasco, M. A. Pantaleo

**Affiliations:** 1 Department of Specialized, Experimental, and Diagnostic Medicine, S. Orsola-Malpighi Hospital, University of Bologna, Bologna, Italy; 2 Department of Radiology, S. Orsola Malpighi Hospital, Bologna University, Bologna, Italy; 3 Centro Diagnostico Italiano, Reparto Cyberknife, Milan, Italy; 4 Radiation Oncology Unit, Bellaria Hospital, Bologna, Italy; 5 Department of General and Emergency Surgery and Organ Transplantation, S. Orsola-Malpighi Hospital, University of Bologna, Bologna, Italy; 6 Interdepartmental Centre of Cancer Research “G. Prodi”, University of Bologna, Bologna, Italy

**Keywords:** Radiotherapy, GIST, Treatment

## Abstract

**Background:**

Gastrointestinal stromal tumor (GIST) is the most common mesenchymal neoplasm of the gastrointestinal tract. The main treatment for localized gastrointestinal stromal tumors is surgical resection. Unresectable or advanced GIST are poorly responsive to conventional cytotoxic chemotherapy but the introduction of tyrosine kinase inhibitors (TKIs) marked a revolutionary step in the treatment of these patients, radically improving prognosis and clinical benefit. Historically GIST has been considered radiation-resistant, and the role of radiotherapy in the management of patients with GIST is currently restricted to symptomatic palliation in current treatment guidelines.

**Case presentation:**

Here we report two patients affected by metastatic GIST, treated with radiotherapy and radiosurgery in combination with TKIs, achieving an unexpected objective response in the first case and a significant clinical benefit associated with a local tumor control of several months in the second case.

**Conclusions:**

These and other successful experiences that are progressively accumulating, open up new scenarios of use of radiation therapy in various settings of treatment. GIST is not universally radioresistant and radiotherapy, especially if combined with molecularly targeted therapy, can improve the outcomes for patients diagnosed with GIST.

## Background

Gastrointestinal stromal tumors (GISTs) are the most common mesenchymal tumors of the gastrointestinal tract. Previously classified as leiomyomas, leiomyosarcomas, leiomyoblastomas or schwannomas, they are now recognized as a distinct entity, arising from the interstitial cells of Cajal or their precursors, specialized pacemaker playing a critical role in the coordination of normal motility within the gastrointestinal tract.

GIST molecular pathophysiology is a mutation-driven process, in most cases (85–90%) a gain-of-function KIT gene mutations, which lead to constitutive activation of KIT kinase activity and to uncontrolled cell proliferation. A notably smaller proportion (5–8%) is associated with analogous mutations in platelet-derived growth factor receptor α (PDGFRα) and <10% contain no identified receptor tyrosine-kinase mutations, referred as KIT/PDGFRA wild-type GISTs [1–4].

The advent of the tyrosine kinase inhibitors has dramatically revolutionized the therapeutic approach to gastrointestinal stromal tumor and improved the outcome of these patients, becoming the standard systemic therapies for locally advanced/metastatic GIST [5, 6].

Most patients obtain good, durable responses to treatment; nevertheless, almost the great majority of patients in a metastatic setting develop resistance, leading to failure of tyrosine-kinase inhibitors and bringing the clinicians to consider an increasingly wide spectrum of loco-regional treatment options.

The decisions are based on the specific clinical history of each patient and have the aim of maximizing the duration of each therapeutic method and, ultimately, the overall sequential treatment strategy.

Historically GIST has been considered radiation-resistant, and radiotherapy is recommended only for palliative purpose of bone metastases in current treatment guidelines [7].

However, some experiences accumulated in recent times, including ours, suggest that GIST metastases are moderately radiosensitive, and frequently stabilize for several months with radiotherapy.

Radiotherapy appears to be a well-tolerated treatment that should be considered in the management of metastatic GIST not only with palliative purposes but, in our opinion, even in other settings of treatment.

In this article, we report the strategy of integration between radiotherapy and medical treatment, focusing on new potential scenarios not explored yet, that may enlarge the treatment opportunities for these patients.

## Cases presentation

### Case 1

In October 2008 a 62 years old male underwent urgency total gastrectomy due to a massive hematemesis. Histological examination revealed a CD 117 positive, DOG 1 positive gastric GIST with high risk of recurrence according to Miettinen classification (6 cm of diameter, 15 mitoses per 50 high power field). No tumor rupture was observed.

In January 2010, a CT scan documented the appearance of multiple liver metastases, thus the patient was enrolled in CAMN107G2301 trial and a first line therapy with imatinib mesylate at a daily dose of 400 mg was started. The treatment was pursued regularly, with good tolerance and stability of disease until July 2011, when a mild increase in size of hepatic lesions led to stop the trial and an imatinib dose escalation to 800 mg/day was started.

In May 2012 a new solid paracaval lesion with thrombosis of the inferior vena cava was identified (diameter 27 × 45 mm), and therapy was switched to a second line treatment with oral sunitinib at the dose of 37.5 mg/day.

A CT scan after 5 months of sunitinib treatment showed progression in the size of the paracaval lesion (diameter 130 × 110 mm) with compression of the vena cava and coeliac trunk, therefore we discontinued sunitinib and prescribed regorafenib 160 mg/day (21 days on, 7 days off). The mass became quickly symptomatic with local pain and hiccup. A surgical consultation excluded reintervention because of the site of disease, inseparable from the great vessels, and because of the sequelae of previous surgery.

In view of the symptoms development, of the rapid increase in size of the mass (about 8 cm in few months) and in absence of surgical options, in November 2012 we administered external radiotherapy combined with regorafenib at a dose of 160 mg/day.

**Fig. 1:**
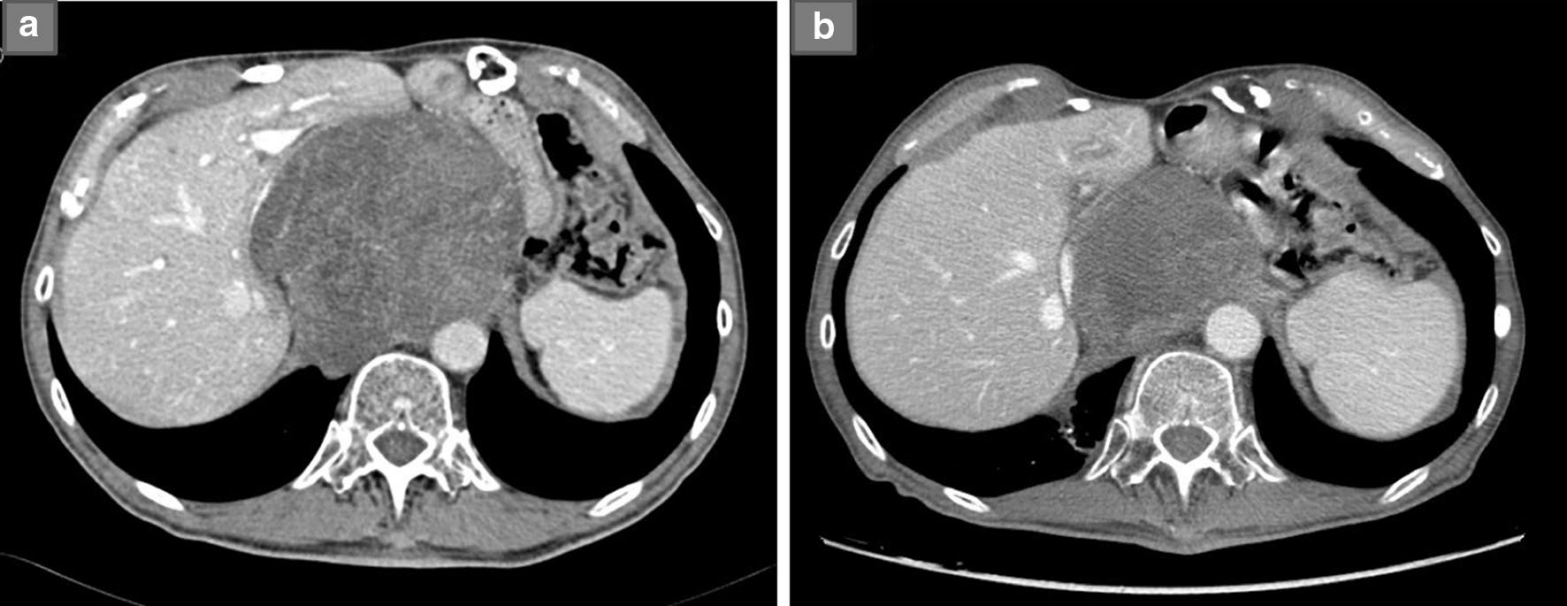
**a** CT scan documenting a voluminous GIST metastasis compressing the vena cava (diameter 130 × 110 mm). **b** CT scan performed 4 months after radiotherapy showing significant tumor shrinkage

A dose of 35 Gray was administered in 14 sessions, with good subjective tolerance by the patient and without complications, obtaining an objective response, with decrease in size of the paracaval mass from 130 to 80 mm and clinical benefit on pain (Fig. 1).

Despite a regorafenib dose reduction for toxicity (120 mg/day—21 days on, 7 days off schedule) the patient has maintained a stable disease, both in the liver and in the radio-treated mass, for over three years, until now.

### Case 2

In January 2012, a 44 years-old male diagnosed with gastric GIST with multiple liver metastases and previously treated with imatinib and sunitinib was referred to our institution.

Clinically the patient complained a number of symptoms related to compression of the tumor, mainly nausea, vomiting and abdominal pain.

Because of the clinical picture, the young age and the lack, at that time, of approved third-line therapies, we decided for a debulking surgery.

In March 2012 a partial gastrectomy and right hepatectomy were performed, histology confirming the diagnosis of GIST with a high mitotic index (mitoses 30/50 high power field). The surgical approach was no radical, with residual hepatic disease, thus, after post-operative recovery, a medical treatment with imatinib 400 mg/day was resumed, subsequently increased to 800 mg/day for increase in size of liver metastases.

In October 2013 a CT scan documented the appearance of a new lesion below the diaphragm in the right pararenal (diameter 34 mm) associated with significant pain, poorly responsive to opioids.

**Fig. 2:**
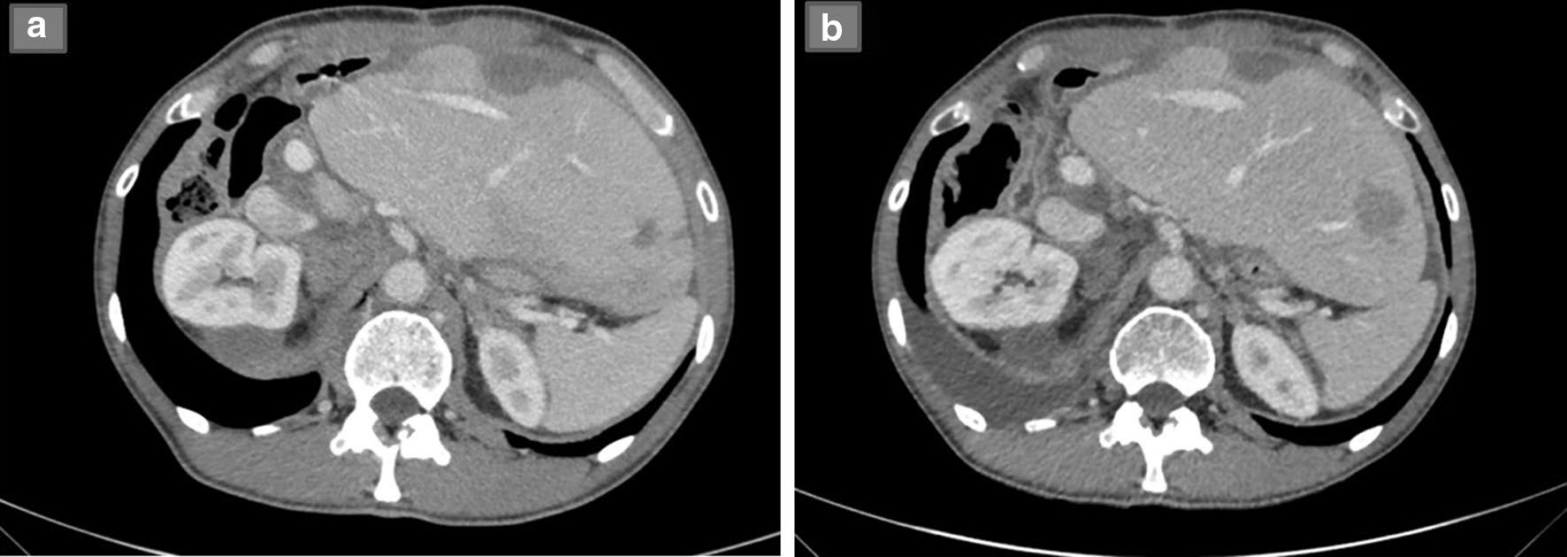
**a** CT scan documenting right pararenal hypervascular metastatsis (diameter 34 mm). **b** CT scan performed after cyberknife. The lesion, substantially stable in size, became completely hypodense

Considering the exclusively focal progression of disease and the symptoms development we decided for local radiosurgery: a cyberknife treatment was performed (at first 4500 cGy delivered in 5 sessions; after 60 days repeated a second dose of 4000 cGy fractionated in 4 sessions), well-tolerated, burdened only by nausea which required a discontinuation of imatinib during sessions. Radiological assessment after cyberknife showed disease stabilization without shrinkage of tumor size, but with a decrease in hypervascularity of the mass, and the patient referred an early improvement in pain (Fig. 2). Few months later the patient developed a critical left supraclavicular mass (diameter 46 × 37 mm), progressively increasing and displacing the trachea, causing worsening pain, hacking cough and dysphagia. In this phase we decided for a combination strategy, integrating medical treatment with sunitinib rechallange with a cyberknife treatment of the supraclavicular mass (total dosage of 32 Gy in 5 sessions). Also in this case, in addition to a dimensional stabilization of the lesion, the patient obtained a substantial clinical benefit.

Unfortunately, in the following months, our patient exhibited a rapid disease progression, whilst maintaining a dimensional stability of the radiosurgical-treated masses (for about 5 months) and a prolonged symptoms control.

## Discussion

Although the role of radiation therapy is well established for the treatment of soft tissue sarcomas, GIST has been historically considered radioresistant [8, 9], and the use of this method has been discouraged mainly because of the elective abdominal pattern of spread, which require too large fields of irradiation, often resulting in unacceptable bowel toxicity.

Based on these considerations, currently the use of radiation therapy in GIST is rarely considered and is recommended only for the palliative treatment of bone metastases [7].

However, to date some publications suggest the efficacy of radiation therapy in the management of GIST [10–12].

Herein we report two very peculiar cases suggesting a possible successful use of radiotherapy as an integrated approach in GIST management, to obtain a long lasting disease stabilization, and, in selected cases, even objective responses.

In the first case we combined radiotherapy with regorafenib for the treatment of a voluminous, progressively increasing and symptomatic paracaval lesion, obtaining not only a symptoms control but also a significant tumor shrinkage for three years.

The second case, instead, is the first published experience, to our knowledge, of cyberknife treatment in GIST. Cyberknife is a device that combines a robotic arm with a linear accelerator, has been developed for stereotactic radiotherapy and may provide an adequate dose to the tumor while decreasing the dose to the surrounding normal tissue. Cyberknife was administered in both a pararenal and a supraclavicular mass, at different times, and resulted in a well-tolerated treatment which provided local control of the masses and rapid improvement of the symptoms.

Currently the standard of care for resectable GIST is surgery, followed by adjuvant imatinib for high risk recurrence. Inoperable or metastatic tumors are treated primarily with imatinib, which should be pursued indefinitely until the development of progression that occurs, generally, after a median time of 24 months [13].

For patients who progress to imatinib, sunitinib is an effective treatment option and currently represent the only proven second-line therapy for advanced GISTs [14]. However, due to the development of resistance to both of these two drugs in the majority of patients, the need for third-line therapy arose. Based on the phase III placebo-controlled GRID trial, regorafenib, an oral multi-targeted inhibitor with activity against multiple kinases including KIT, RET, RAF1, BRAF, PDGFR, FGFR and angiogenesis (VEGFR), has shown an increase of median PFS (4–8 vs 0–9 months in regorafenib group, p < 0.0001)and is now established as the third-line therapy of metastatic GISTs [15].

In recent years the development of three lines of therapy has improved a lot the prognosis and survival of patients with GIST, making more and more evident the need not only of further lines of therapy, but, above all, the necessity of multidisciplinary approaches in order to maximize the clinical benefit and the duration of treatment with each single agent. In this context we invite to consider much more the role of radiation therapy in GIST, which could prove to be invaluable, as it has been for the radiofrequency ablation of liver metastases, now definitively imposed as a successful option in providing effective local tumor control, especially if combined with TKIs therapy [16, 17].

There are several scenarios in which radiation therapy may provide a potential benefit:

I. radiotherapy of metastases for palliation of local symptoms with low toxicities, a possibility already widely explored
II. radiotherapy of focally progressing lesions, with the aim of overcoming emergent resistant clones, not only with a palliative intent but also cytoreductive, in combination with systemic therapy which continue acting on the sensitive clones
III. in the peri-operative setting, combined with imatinib, especially for GIST at high risk of local recurrence, where surgery is often demolitive, such as rectal and esophageal GIST
IV. definitive radiotherapy alternative to surgery in localized GIST in elderly patients with comorbidities or in case of unresectable tumors

In the largest published series, Joensuu et al. presented a phase II prospective study of 25 patients affected by liver, soft-tissue, intra-abdominal and bone GIST metastases, treated with external beam radiotherapy, at a cumulative dose of approximately 40 Gy, whilst maintaining systemic therapy during irradiation (11 patients continued to receive imatinib, 4 sunitinib, 2 nilotinib, 1 regorafenib and 1 a combination of sorafenib and everolimus). Two patients achieved partial remission, 20 patients had a durable stabilization of the target lesion, with a median duration of stabilization of 16 months, and only 3 progressed (9).

Obviously it is difficult to determine if the systemic therapy is a confounding factor in assessing the response to a local treatment and if it can increase sensitivity to radiotherapy. Realistically the efficacy achieved could result rather from the combined effects of radiotherapy and tyrosine kinase inhibitors, and might be less with radiation alone.

Some studies suggest that imatinib can increase the radiosensitivity in cell lines, specifically human glioma and anaplastic thyroid cancer cell lines. In particular, imatinib can reduce the levels of cellular RAD51, an essential component of the homologous DNA repair pathway implicated as a determinant of cellular radiosensitivity, leading to increased radiosensitivity in vitro and in vivo without undue toxicity toward normal cells and tissues [18, 19].

Ciresa et al. described a successful use of neo-adjuvant radiotherapy in a case of rectal GIST extending to the anal canal, who received imatinib 400 mg/day and concomitant radiotherapy (total dose 50.4 Gy in fractions of 1.8 Gy daily) obtaining a partial clinical response 3 weeks after the end of combination treatment. The patient then underwent a sphincter-saving surgical procedure, with a complete pathological response at the histological examination [20].

Although there are no prospective data that produce this evidence, radiotherapy may be considered, associated with TKI and preferably in the context of clinical studies, in the treatment of GIST and in particular of those in the rectal, esophageal and duodenal site, to evaluate the effectiveness of this approach in both the down-staging and the prevention of local recurrence as well as for the epithelial counterpart, even though it is well known the histology is completely different.

Regarding the cyberknife, it is an image-guided frameless robotic technology for whole-body radiosurgery, used for classic single-fraction radiosurgery and for hypofractionated treatments, which treat tumors with supreme accuracy, essentially “painting” the mass with radiation and sparing surrounding healthy tissue.

Unlike some radiosurgery systems, which can only treat primary or secondary brain neoplasms, the cyberknife may be applicated to extracranial use and has been tested in a broad range of tumors, including prostate, lung, spine, liver, pancreas, and kidney. However, to our knowledge, no experience has so far been published regarding the application of the cyberknife in GIST.

This radiosurgery strategy has emerged as an additional tool in oncology armamentarium, extremely attractive for the high tumor control, the low toxicity and the repeatability of the treatments for recurrent metastases [21, 22].

Optimal management of patients diagnosed with GIST is multidisciplinary, should be performed only at specialized oncological center, and requires strong cooperation among different expertises, including oncology, surgery, ultrasound, radiology, radiotherapy, radiosurgery and interventional radiology.

It is not excluded that in the very near future, not only radiation but also other approaches such as arterial embolization, cyber-knife and HIFU ablation (high intensity focused ultrasound) can carve out a role in the treatment of this tumor, increasingly articulate and complex.

## Conclusions

To date, the evidence regarding the use of radiation therapy in GIST is limited to small case series, therefore, the impression emerging from these experiences is that this approach is effective not only in a strictly palliative field, but may also provide objective responses and long-term disease control in selected patients, without any significant toxicity or impact on quality of life.

We decided to report these two cases because, in our view, are very interesting in several aspects: the unexpected significant objective response obtained with the combination of regorafenib and radiotherapy in the first case, and the experience with the technique of cyberknife in the second case, which we hope could find an increasingly wide range of use in the immediate future.

We can certainly conclude that radiation therapy, unjustifiably underutilized until now, should be considered a viable option in the treatment of locally advanced\metastatic GISTs. Nevertheless further studies are needed to standardize its correct role in the different settings of treatment, but also the effective dosage and the irradiation techniques.

## Abbreviations

CT: computed tomography
GIST: gastrointestinal stromal tumor
PDGFRα: platelet-derived growth factor receptor α
TKIs: tyrosine kinase inhibitors
